# Some Points about Artificial Palates

**Published:** 1897-05

**Authors:** Oscar Boort

**Affiliations:** Tabor, Iowa.


ARTICLE VIII.

### SOME POINTS ABOUT ARTIFICIAL PALATES.

BY OSCAR BOORT, D. D. S., TABOR, IOWA.

In the construction of artificial palates it should always be borne in mind that the levator palatai muscles are fully developed and in active operation. The cleft is the result of arrested development of the naso-pharyngeal valve and the palate bones, variably extensive. Movement of the levator muscles took place in the act of deglutition and articulation, the same as in the normal velum. At every nasal tone the levators drop, and with every attempt at throat or chest tones they rise, as though never weary of performing a useless task. Every successful effort to correct the defect with a mechanical appliance must comprehend these facts, and be so constructed that the levator muscles can take hold of and move it when they move.

With this clearly in mind it will at once be seen that a substance like elastic rubber will in nowise answer the requirements in an artificial velum; instead of being moved up and down its edges curl up and the levators have no control over it; besides, elastic rubber in the mouth soon deteriorates and becomes filthy. Whatever the substance used in artificial vela it should be hard and capable of taking a high polish. The velum must be light and so formed as to lie between and over the levator palati muscles, and so attached to the supporting plate that it will swing freely from its posterior border, and be constrained by a delicate spring in such a manner as to easily yield to the muscular action and be returned to its normal position when the muscles are at rest.

In gold alone, or in gold and hard rubber combined, may be found the proper materials for such work. To make the velum sufficiently light, whether of hard rubber or gold, it must be made hollow. Usually a congenital cleft is not less extensive than an entire bifurcation of the soft palate, but occasionally the cleft does not reach the border of the palate bones. Then it will be necessary to use the lance and carry the bifurcation forward in the median line to the posterior border of the palate bones, to make room for the appliance.

Congenital clefts are uniform in character and vary only in extent, but accidental lesions of the palate are irregular in character and must be treated accordingly. When disease, such as syphilis, has destroyed the velum and left the levator muscles untouched, however extensive its ravages in other parts, speech may be restored the same as in congenital cleft, with the advantage that the restoration will be complete and perfect at once, while in the congenital cleft practice alone will lead to perfection. The artificial velum, in syphilitic cleft, should always be made of a non-corrosive metal like gold or platinum, since rubber is liable to irritate the parts by keeping them unnaturally heated.

If the levator muscles are destroyed, of course no movable velm can be supplied; yet great benefit to speech will be obtained by extending the plate back just far enough that in the act of deglutition the superior constrictor of the pharynx will meet and touch it.—*Dental Digest.*

---

